# Neutralization Sensitivity of HIV-1 CRF07_BC From an Untreated Patient With a Focus on Evolution Over Time

**DOI:** 10.3389/fcimb.2022.862754

**Published:** 2022-03-17

**Authors:** Lijie Wang, Shujia Liang, Jianhua Huang, Yibo Ding, Lin He, Yanling Hao, Li Ren, Meiling Zhu, Yi Feng, Abdur Rashid, Yue Liu, Shibo Jiang, Kunxue Hong, Liying Ma

**Affiliations:** ^1^ State Key Laboratory of Infectious Disease Prevention and Control, National Center for AIDS/STD Control and Prevention, Collaborative Innovation Center for Diagnosis and Treatment of Infectious Diseases, Chinese Center for Disease Control and Prevention, Beijing, China; ^2^ Guangxi Key Laboratory of AIDS Prevention and Control and Achievement Transformation, Guangxi Center for Disease Prevention and Control, Nanning, China; ^3^ Hengzhou Center for Disease Prevention and Control, Hengzhou, China; ^4^ Department of Biochemistry and Molecular Biology, Bloomberg School of Public Health, Johns Hopkins University, Baltimore, MD, United States; ^5^ Key Laboratory of Medical Molecular Virology of Ministry of Education/ National Health Council/Chinese Academy of Medical Sciences, School of Basic Medical Sciences, Shanghai Institute of Infectious Disease and Biosecurity, Fudan University, Shanghai, China

**Keywords:** HIV-1, CRF_07BC, envelope, neutralization sensitivity, evolution, long-term non-progressor

## Abstract

The diversity of HIV-1 envelope (Env) glycoproteins affects the potency and breadth of broadly neutralizing antibodies (bNAbs), a promising alternative to antiretroviral drugs for the prevention and treatment of HIV-1 infection. To facilitate immunogen design and development of therapeutic neutralizing antibodies, we characterized viral evolution and monitored the changes in neutralizing activity/sensitivity of a long-term non-progressor patient with HIV-1 CRF07_BC infection. Fifty-nine full-length Env gene fragments were derived from four plasma samples sequentially harvested from the patient between 2016 and 2020. Sequencing of patient-derived Env genes revealed that potential N-linked glycosylation sites (PNGS) in V1 and V5 significantly increased over time. Further, 24 functional Env-pseudotyped viruses were generated based on Env gene sequences. While all 24 Env-pseudotyped viruses remained sensitive to concurrent and subsequent autologous plasma, as well as bNAbs, including 10E8, VRC01, and 12A21, Env-pseudotyped viruses corresponding to later sampling time were increasingly more resistant to autologous plasma and bNAbs. All 24 Env-pseudotyped viruses were resistant to bNAbs 2G12, PGT121, and PGT135. The neutralization breadth of plasma from all four sequential samples was 100% against the global HIV-1 reference panel. Immune escape mutants resulted in increased resistance to bNAb targeting of different epitopes. Our study identified known mutations F277W in gp41 and previously uncharacterized mutation S465T in V5 which may be associated with increased viral resistance to bNAbs.

## Introduction

HIV-1 is a rapidly mutating RNA retrovirus that integrates into the host genome within about 72 h after infection. Once integrated, it forms a latent reservoir of infected cells to resist elimination of the immune system (Anderson and Maldarelli, 2018). HIV-1 infection is usually initiated by a single or small number of transmitted/founder (TF) viruses. Nonetheless, varying levels of antibody and T-cell responses are reported to form the evolutionary pressure that contributes to the viral diversity within each infected person (Burton et al., 2012; Leda et al., 2020). Therefore, HIV-1 has been rapidly spreading with increasing global diversity since its discovery 40 years ago, making HIV/AIDS a major health and socioeconomic burden worldwide.

HIV-1 antibodies that bind to the natural envelope (Env) trimer exert a neutralizing effect by blocking the Env-CD4 receptor or blocking fusion with host cell virions (Burton and Mascola, 2015). Broadly neutralizing antibodies (bNAbs) can bind to Env trimers on virions of even Tier 2 viruses, thus effectively neutralizing a large proportion of HIV-1 infection primary strains. However, one report showed that only 10% of HIV-infected individuals have developed plasma neutralization for approximately 90% of HIV-1 global strains over several years of infection (Hraber et al., 2014). Meanwhile, 5% to 15% of HIV-infected patients can naturally maintain high CD4+ T-cell counts and remain asymptomatic for several years without antiretroviral therapy (ART). These so-called long-term non-progressors (LTNPs) (Grabar et al., 2009) are associated with the production of bNAbs (Gonzalez et al., 2018). Hundreds of bNAbs targeting different epitopes were isolated from adults and children (McCoy and Burton, 2017), and even more than one bNAb lineage can appear in the same individual (Wibmer et al., 2017; Ditse et al., 2018). Although these antibodies target different epitopes of the HIV-1 Env trimer, the virus can escape immune recognition of these antibodies through conformational masking, glycosylation, or sequence variability (Chuang et al., 2019; Berndsen et al., 2020). So far, immunization with relatively conserved sites of vulnerability on HIV-1 Env does not induce highly potent bNAbs. In addition, the etiological features linked to the inducement of bNAbs or the mechanism of virus immune escape in the presence of bNAbs remains enigmatic.

Understanding the viral characteristics related to the development of bNAbs in HIV-1 natural infection, especially the critical events involved in the evolution of virus Env and bNAb lineage, may provide valuable information for the successful design of an HIV-1 vaccine regimen that induces full maturation of the bNAb lineage. Here, based on 4 sequential specimens sampled between March 2016 and September 2020, we conducted an etiological and immunological investigation on a long-term non-progressor with HIV-1 CRF07_BC infection. Results demonstrated that 1) neutralization sensitivity was associated with evolution of the targeted domain of neutralization, not the entire envelope protein, 2) immune escape mutants gained greater resistance to bNAbs targeting different epitopes, and 3) the site (residue 465 in V5) and mutation (F277W in gp41) may be associated with immune escape.

## Materials and Methods

### Ethics Statement

Ethics approval for this study was obtained from the Ethics Committee of the National Center for AIDS/STD Control and Prevention, Chinese Centre for Disease Control and Prevention (No. X160729429). Patient blood samples were collected after receiving written informed consent.

### Collection and Processing of Biological Samples

At each time point (see **Supplementary Figure 1** for details), 20 ml whole blood was collected in heparin-containing vacutainer tubes (BD, Franklin Lakes, NJ, USA) to prevent clotting. A disposable sterilized pipette was used to dispense 2 ml whole blood and 500 μl each of cryovials, one of which was processed for CD4 counts, and the other three samples were frozen at -80°C. Plasma and peripheral blood monocytes (PBMC) were isolated from 18 ml whole blood by density gradient and frozen at -80°C. All samples were cryopreserved in liquid nitrogen following standard techniques, until further analyzed.

### CD4 Counts and Viral Load Detection

CD4+ T lymphocyte counts were determined from the patients’ whole blood by flow cytometry using BD FACSCalibur (BD, USA), according to the manufacturer’s instructions, and data were analyzed using Multiset™ (BD, USA). A quantitative analysis of HIV-1 RNA in plasma was performed according to the manufacturer’s instructions using Abbott M2000 RealTime HIV-1 assay (Abbott Molecular, Inc., Des Plaines, IL, USA).

### Isolation and Analysis of HIV-1 Strains

HIV-1 strains were isolated from PBMCs by the classical coculture method (Cohen et al., 2018). Viral RNA was extracted from the donor’s plasma, and the nearly full-length genome sequence was obtained by PCR amplification. All amplicons were directly Sanger sequenced, aligned, and analyzed, as previously described (Deymier et al., 2014). HIV-1 co-receptor usage was evaluated using the GHOST cell line, as described previously (Qu et al., 2010). Briefly, GHOST cells stably expressing CD4, as well as co-receptors, either CXCR4 or CCR5, were seeded in a 24-well plate at a density of 1 × 10^5^ cells/well (0.5 ml/well). GHOST cells were infected with virus stocks (200 μl/well) at 60%–70% confluency. Green fluorescent protein (GFP) expression levels of infected cells were detected by flow cytometry to determine the utilization of co-receptors at 48 h postinfection.

### Pseudovirus-Based Neutralization Assay *In Vitro*

A neutralization assay against heterologous/homologous HIV-1 pseudoviruses was performed, as previously described (Kong et al., 2018). Briefly, heat-inactivated plasma samples or bNAbs were incubated with single-round competent pseudoviruses. TZM-bl cells containing dextran hydrochloride (DEAE) were added into a 96-well flat-bottom culture plate and incubated for 48 h at 37°C. Negative control was set as TZM-bl cells not exposed to the virus. Positive control consisted of pseudotyped viruses incubated with TZM-bl cells. To quantify reductions in virus infection in TZM-bl cells, Tat-regulated firefly luciferase reporter gene expression was used. Neutralization titers were measured with a luminometer and reported as relative luminescence units (RLUs) (PerkinElmer, Waltham, MA, USA). Either 50% inhibitory dose (ID50) of plasma or 50% inhibitory concentration (IC50) of bNAbs was calculated. All neutralization experiments were done at least twice.

### Viral RNA Extraction, cDNA Synthesis, and Single-Genome Amplification

The detailed process was performed as previously described (Sha et al., 2020). In short, viral RNA was extracted from plasma samples using the QIAamp Viral RNA Mini Kit (Qiagen, Germany), as described in the manufacturer’s protocol. The first-strand cDNA was immediately synthesized by the SuperScript First-Strand Synthesis System (Invitrogen, Carlsbad, CA, USA) in accordance with the manufacturer’s instructions. Nested PCR was performed by serially diluted cDNA for the Env gene. The amplified Env would be sequenced when positive amplification was less than 30% in all diluted cDNA of each sample. All PCR reactions were performed using PrimeSTAR Max DNA Polymerase (Takara, Shiga, Japan).

### Sequence Alignment and Phylogenetic Analyses

The full-length envelope sequences were assembled and edited using the Sequencher Program v4.10.1 (Gene Codes, Ann Arbor, MI, USA). Sequences were aligned using Gene Cutter (http://www.hiv.lanl.gov/content/sequence/GENE_CUTTER/cutter.html) and then manually edited using BioEdit. The length of the V1, V2, V3, V4, and V5 variable regions was analyzed at Variable Region Characteristics (https://www.hiv.lanl.gov/content/sequence/VAR_REG_CHAR/index.html), and potential N-linked glycosylation sites (PNGS) were predicted by NetNGlyc 1.0 Server. Mega X was used for genetic distance calculation. Co-receptor tropism was detected using the Web-based geno2pheno software.

### Pseudovirus Preparation, Titration, and Neutralization Sensitivity Assays

The Env single-genome amplification (SGA) PCR products were cloned into the vector pcDNA™ 3.1 Directional TOPO^®^ Expression Kit (Invitrogen, USA), which allows the Env gene to be inserted in the correct orientation with a cytomegalovirus promoter for protein expression (Hu et al., 2021). Pseudoviruses were prepared and titrated, as described previously (Zhang et al., 2019). Briefly, 293T/17 cells were co-transfected with the Env/Rev expression plasmid and an Env-deficient HIV-1 backbone vector (pSG3ΔEnv), using Lipofectamine™ 3000 Transfection Reagent (Invitrogen, USA), and the cultures were incubated for 48 h at 37°C. Obtained pseudoviruses were filtered through a 0.45-μm filter and frozen at −80°C. The 50% tissue culture infectious dose (TCID50) of the pseudoviruses was assessed by preparing serial 11-fold dilutions of the pseudovirus. TZM-bl cells containing DEAE were added to the virus, and a negative control was set as TZM-bl cells left untreated with the virus. Following incubation at 37°C, 5% CO_2_, for 48 h, viral infectivity was measured as RLUs using a luminometer. The infective dose that yielded ≤50,000 RLUs was used to test neutralization sensitivity to autologous plasma or bNAbs. Neutralization sensitivity assay was performed as described above.

### Analysis of bNAbs Sensitivity Signatures of Epitope

Under the immune pressure of neutralizing antibodies, viruses escape through mutation on the neutralizing epitopes, resulting in resistance to bNAbs. To explain such resistance, we analyzed the variation of Env sequences containing resistant amino acids of key signature sites at different time points. Sequence logos were drawn in WebLogo (http://weblogo.berkeley.edu/logo.cgi).

### Statistical Analysis

R software (version 3.6.3) was used to perform statistical analyses, and graphical representations were generated with GraphPad Prism software (version 5.0). One-way analysis of variance (ANOVA) was used for group comparison with homogeneous variances, but a non-parametric test (Kruskal–Wallis) for non-homogeneous variances. Bonferroni correction for multiple comparisons was applied to all p-values. p < 0.05 was considered statistically significant.

## Results

### Study Subject

The patient, male, was born in 1973 and started injecting drug use in 1997. He was diagnosed as HIV-positive in March 2001 with ELISA and WB methods and had never received antiviral treatment. The patient started Methadone Replacement Therapy in 2011.

To evaluate the viral load and CD4 counts in the patient, whole blood and plasma samples were collected multiple times between 2010 and 2020 (**Supplementary Figure 1**). Despite being diagnosed HIV-positive for over 20 years, his CD4 counts remained at a high level, ranging from 692 to 934 cells/µl. The viral load of the patient only marginally increased from 14,700 copies/ml in 2016 to 35,000 copies/ml in 2020.

### Biological and Genetic Characterization of Patient-Derived HIV

The virus was isolated from whole-blood specimen sampled in 2016, and the HIV-1 strain was derived with viral titer of 15,000 TCID50/ml measured in TZM-bl cells. Co-receptor detection showed that it was a CCR5 tropic HIV-1 strain (**Supplementary Figure 2**). Analysis of the nearly full-length sequence showed that the donor was infected with the HIV-1 CRF07_BC subtype (**Supplementary Figure 3**), most likely composed of four C fragments and three B fragments (**Supplementary Figure 4**).

### Phylogenetic Analyses of the Envelope Genes

**Figure 1:**
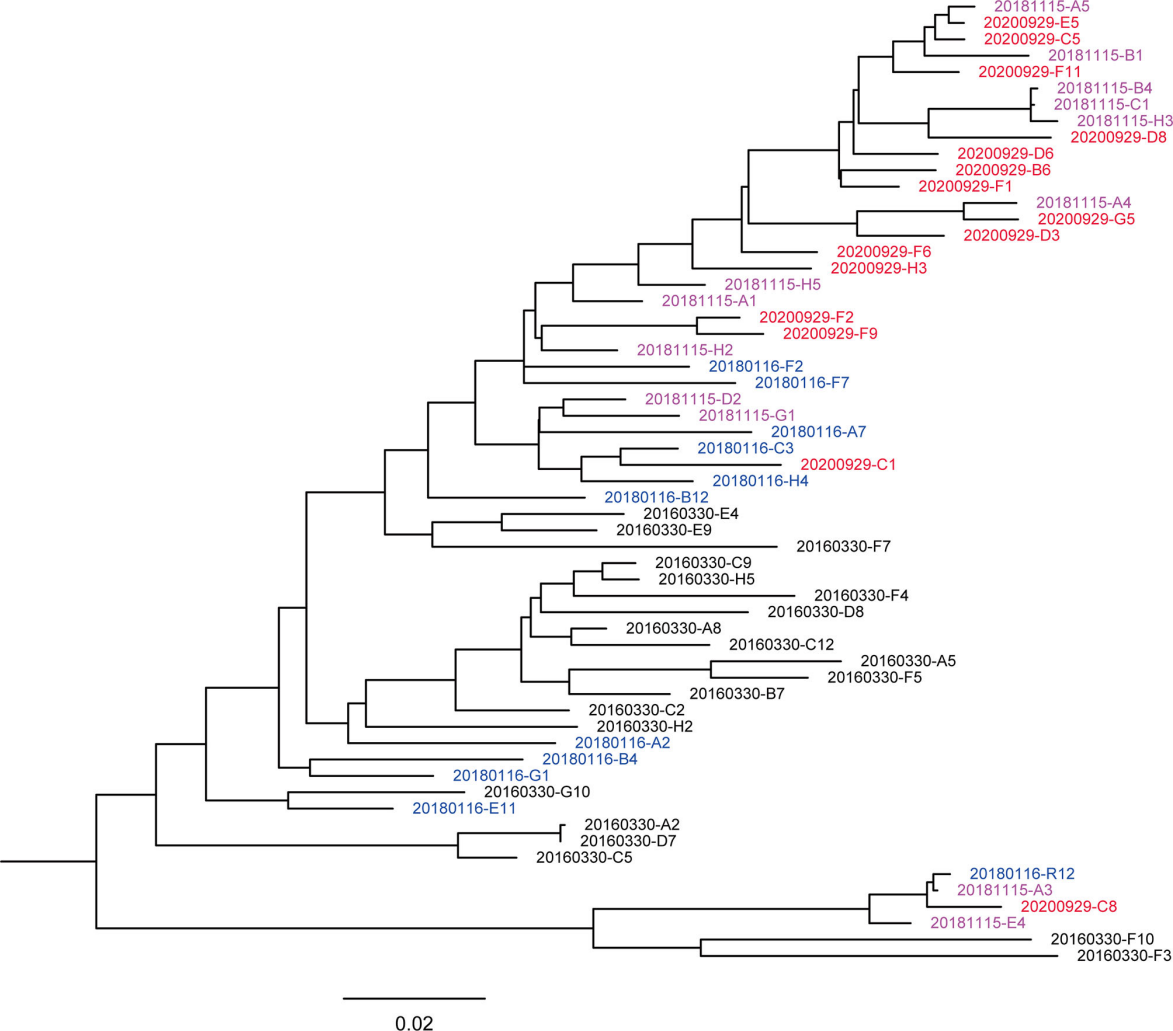
Maximum likelihood method-based phylogenetic tree of the env genes. A total of 59 nearly full-length Env sequences from the donor. The phylogenetic tree was inferred with IQtree v1.6.1 and visualized with FigTree. Tree showed that the sequences intermingled, crossing different time points. The sequences from different sampling time points are highlighted in black (201603), blue (201801), purple-pink (201811), and red (202009), respectively. The bar under the phylogenetic tree is the genetic distance scale.

A total of 59 nearly full-length Env sequences were obtained from 4 sequential plasmas by SGA, with 20, 11, 13, and 15 sequences obtained from 201603, 201801, 201811, and 202009, respectively. Seven of these sequences showed premature termination codons (2 in 201603, 2 in 201801, 2 in 201811, and 1 in 202009), indicating the presence of defective Env in these sequences. Sequences were aligned, and a maximum likelihood phylogenetic tree was inferred with IQtree v1.6.1 (**Figure 1**). The phylogenetic tree showed that the sequences intermingled, crossing at different time points. However, in the evolutionary trend, the sequences still formed relaxed time-specific lineages.

Viral quasispecies showed different degrees of variation at each time point and formed a few secondary evolutionary clusters on the phylogenetic tree, indicating that they evolved in different directions under host-specific selection pressure. The genetic distances of sequences at 201603, 201801, 201811, and 202009 were 0.082 ± 0.006, 0.073 ± 0.006, 0.067 ± 0.005 and 0.057 ± 0.004, respectively. The genetic distance showed a decreasing trend over time (Pearson correlation coefficient r = – 0.509, p < 0.001), suggesting that the sequence diversity of early virus was higher. In addition, the phylogenetic trees showed that a main viral population and a minor viral population were formed in the individual and that they evolved in parallel for more than 4.5 years. Among them, some viral quasispecies at the time point of 201603 in the main viral population disappeared after evolving to 201801, and another small cluster of viral quasispecies became the dominant strains and continued to evolve. Compared with the minor population, the major viral population showed higher diversity, indicating that it reflected better adaptation to host selective pressures. Although the minor population was not eliminated over time, the small number of sequences made further comparison with the biological characteristics of the main population a moot exercise.

### Different Characteristics Among Variable Regions

**Figure 2:**
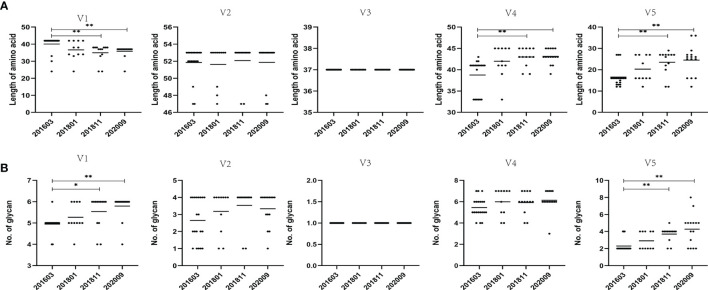
Comparison of amino acid length and PNGS throughout the V1–V5 regions among different sampling data. **(A)** Comparison of amino acid length of the V1–V5 regions, indicating significant differences in amino acid lengths of V1, V4, and V5 among different time points. **(B)** Comparisons of glycan number of the V1–V5 regions, showing significant differences in glycan number of V1 and V5 among different time points. Representative data are shown (*p < 0.05; **p < 0.01).

To assess viral evolution in the patient, amino acid sequence length and PNGS in the five variable regions of the Env gene were compared among samples harvested at four time points (**Figures 2A, B**). By measuring the length of amino acid sequences, it was observed that the amino acid sequence at each time point of V2 and V3 remained stable, whereas the amino acid sequence at each time point of the V4 and V5 regions showed an increasing trend over time, compared with the 201603 time point (p < 0.01). It is noteworthy that the amino acid sequence of the V1 region showed a decreasing trend over time, compared with that of the 201603 time point (p < 0.01).

The LANL HIV database Consensus Maker tool was used to construct the consensus sequence of the 59 Env sequences, followed by analysis of NetNGlyc 1.0 Server with 7 PNGS. At different time points, more PNGS were found in the V1 and V4 regions, with a median range of 5 to 7, possibly associated with a significant degree of variation from base to base. The PNGS in V1, V2, V4, and V5 showed an increasing trend over time, but only the increase in PNGS in V1 and V5 was statistically significant (p < 0.01).

These results demonstrated that the genetic distance at later sampling points decreased compared with that of the 201603 sampling point. The difference at each time point was statistically significant (p < 0.05), except the V4 region. In addition, the internal variation at each time point was inconsistent, especially the V1 region at the 201801 sampling point where the genetic distance increased significantly (p < 0.01) and then decreased (**Supplementary Figure 5**).

### Unique Characteristics of the V3 Region

To evaluate the relationship between mutations in the V3 region and biological function of the virus, we further analyzed the V3 loop amino acid mutations and biological features.

The length of the amino acid sequence and the number of PNGS of the V3 region at the 4 sampling time points did not change, and both were very conserved (**Figure 2**). This is consistent with the functional importance of the V3 loop. The genetic distance of the V3 loop at later three sampling time points was significantly and increasingly shorter than the genetic distance of the 201603 time point (p < 0.05). In addition, although the genetic distance of the later three time points had an increasing trend over time, it was not statistically significant. These results could be attributed to the shortening of the sequence of the V1 and V2 regions and lack of significant increase in glycosylation sites, in turn resulting in the exposure of the V3 region to the Env trimer, suggesting that immune pressure is not enough for the virus to escape.

The tetrapeptide of the V3 region, one of the main specific antigenic determinants of GP120, is composed of four characteristic amino acids. In this study, the tetrapeptides of the V3 region are all GPGQ in the 4 sequential samples. Geno2pheno predicted that all four viral sampling points were CCR5-tropic viruses. The high degree of conserved tetrapeptides and co-receptors may be related to the long-term non-progression of the donor’s clinical symptoms.

According to some studies, two key features of the V3 region recognized bNAbs: the conserved GDIR motif and the N332 glycan (Sok et al., 2016; Barnes et al., 2018). Our results demonstrated that 91.53% (54/59) of sequences had the GDIR motif at the base of the V3 loop, 3 sequences carried GNIR motifs, and another 2 sequences had deleted motifs owing to premature termination codons. Fifty-one sequences contained the N332 glycan, and 6 sequences carried the N334 glycan. Another 2 sequences were deleted owing to premature termination codons. Slower and incomplete viral escape resulted in prolonged exposure of the broadly neutralizing epitope, which may, in turn, have aided in maturation of the bNAb lineage.

### Escape of MPER and CD4bs Epitopes Over Time Is Associated With Increased Resistance to bNAbs

**Figure 3:**
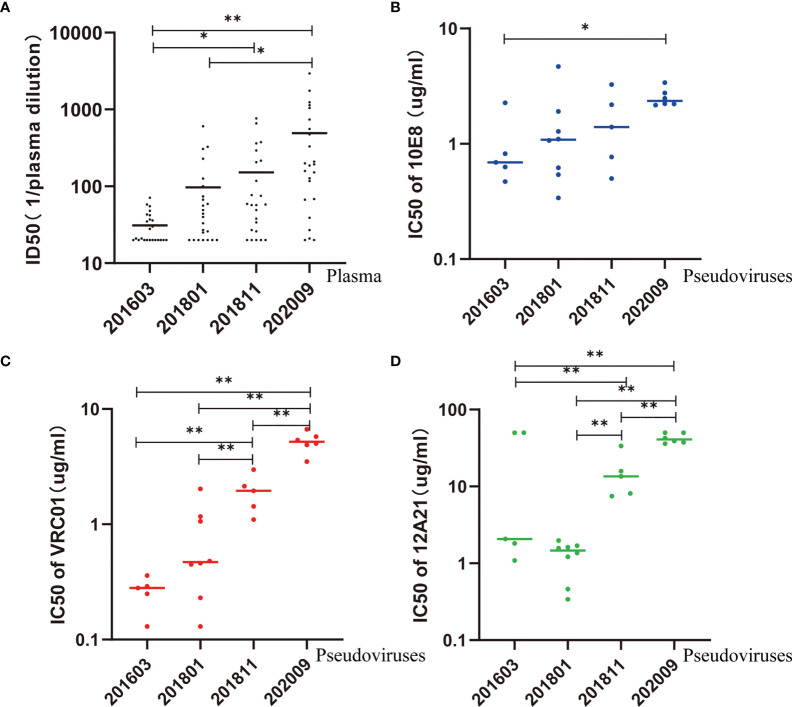
Neutralization sensitivity of pseudovirus to autologous plasma and bNAbs. **(A)** ID50 values of autologous plasma increase over time, suggesting an increase in sensitivity. **(B–D)** IC50 values of 10E8, VRC01, and 12A21 increase over time, suggesting an increase in resistance. Kruskal–Wallis test performed to assess statistical significance. Representative data are shown (*p < 0.05; **p < 0.01).

We constructed 59 vectors of pseudoviruses, each of which contained 59 Env sequences obtained from the sequential samples, whereas 24 of 59 vectors formed infectious pseudoviruses after transfection. The neutralization sensitivity of the 24 Env pseudoviruses was tested against six bNAbs, including the MPER-directed bNAb (10E8), the CD4b-directed bNAbs (VRC01, 12A21), and the V3-glycan region-directed bNAbs (2G12, PGT121, and PGT135) (**Table 1** and **Figure 3**). All pseudoviruses proved to be sensitive to 10E8 and VRC01 with IC50 point estimate values of 1.66 ± 1.13 and 2.00 ± 2.08 μg/ml, respectively. Four (16.67%) pseudoviruses showed neutralization resistance to 12A21 (IC50 > 50 μg/ml); all pseudoviruses showed neutralization resistance to 2G12, PGT121, and PGT135.

**Table 1:** Neutralization sensitivity of pseudoviruses to autologous plasma and bNAbs.

Pseudoviruses	Neutralization of autologous plasma (ID50)				Neutralization of bNAbs (IC50, μg/ml)					
	20160330	20180116	20181115	20200930	10E8	VRC01	12A21	2G12	PGT121	PGT135
201603-1	55	307	374	1138	0.69	0.28	1.81	>50	>50	>50
201603-2	58	326	363	1241	0.63	0.25	1.09	>50	>50	>50
201603-3	30	228	293	741	0.82	0.29	2.07	>50	>50	>50
201603-4	71	603	659	2936	0.47	0.13	>50	>50	>50	>50
201603-5	<20	126	206	557	2.27	0.36	>50	>50	>50	>50
201801-1	21	25	34	158	0.62	0.13	0.34	>50	>50	>50
201801-2	48	74	75	187	1.10	0.48	1.98	>50	>50	>50
201801-3	43	100	216	474	0.34	1.06	1.62	>50	>50	>50
201801-4	<20	67	118	1046	1.91	2.03	1.36	>50	>50	>50
201801-5	<20	27	57	193	0.54	0.46	1.69	>50	>50	>50
201801-6	<20	33	58	152	1.28	0.45	1.57	>50	>50	>50
201801-7	<20	<20	763	1748	1.07	1.17	1.21	>50	>50	>50
201801-8	<20	<20	26	127	4.69	0.23	0.46	>50	>50	>50
201811-1	35	40	38	117	2.18	1.95	8.12	>50	>50	>50
201811-2	42	56	77	199	0.50	1.1	13.49	>50	>50	>50
201811-3	37	59	59	330	0.77	1.43	33.68	>50	>50	>50
201811-4	21	49	49	209	1.40	2.14	15.84	>50	>50	>50
201811-5	<20	<20	<20	68	3.27	2.98	7.49	>50	>50	>50
202009-1	36	44	57	67	3.40	6.68	42.59	>50	>50	>50
202009-2	28	26	27	39	2.76	5.02	37.52	>50	>50	>50
202009-3	<20	<20	<20	21	2.48	5.76	38.91	>50	>50	>50
202009-4	<20	<20	<20	27	2.17	3.5	35.91	>50	>50	>50
202009-5	<20	<20	<20	<20	2.22	4.89	>50	>50	>50	>50
202009-6	<20	<20	<20	<20	2.23	5.39	>50	>50	>50	>50

In comparison, the IC50 of 10E8 was significantly higher in pseudovirus 202009 than that in pseudovirus 201603 (p < 0.05) (**Figure 3B**). Additionally, the IC50 of VRC01 or 12A21 was significantly higher in pseudovirus 202009 than that in pseudoviruses 201811, 201801, and 201603 (all p < 0.01), or in pseudovirus 201811 compared to that in pseudoviruses 201801 or 201603 (both p < 0.01) (**Figures 3C, D**), indicating that the neutralization sensitivity of VRC01 and 12A21 to pseudoviruses had decreased over time.

**Figure 4:**
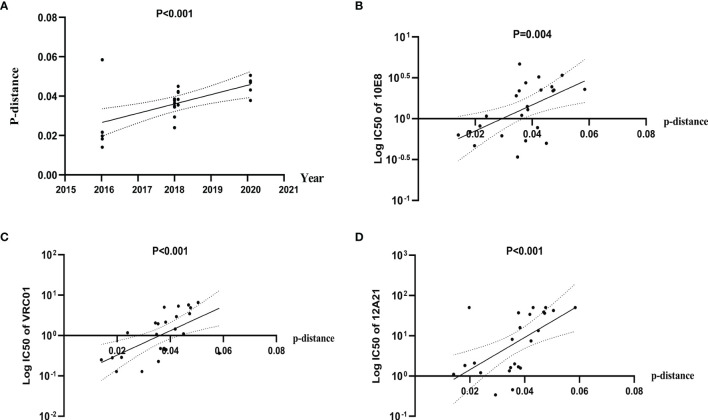
Diversification of Env pseudovirus variants, over a period spanning 2016 to 2020, is associated with increased resistance to bNAbs. **(A)** Genetic distance between consensus sequence and pseudovirus sequence correlates with the calendar year and increases over time. Evolutionary divergence was estimated by distance matrix using MEGA software (Pearson correlation). **(B–D)** IC50 values of 10E8, VRC01, and 12A21 were positively correlated with distance, indicating an increase in resistance (Spearman correlation).

To explore the possible influence of genetic diversity on bNAbs sensitivity, we analyzed the correlation between IC50s to bNAbs and divergence of the pseudoviruses. We observed a significant positive correlation of divergence over time (Pearson correlation, p < 0.0001) (**Figure 4A**), indicating a diversification of pseudovirus variants during the period. Our results also showed a significant positive correlation of divergence with increasing IC50s to bNAbs 10E8 (Spearman correlation, p < 0.0001) (**Figure 4B**) VRC01 and 12A21 (Spearman correlation, p < 0.0001, both) (**Figures 4C, D**). These results demonstrated that genetic distance negatively affects neutralization sensitivity to bNAbs.

In addition, the presence of neutralization resistance signatures indicated that these types of bNAbs may be present in plasma, thus providing information for the isolation of neutralizing antibodies from the donor.

### Evolution of Env Over Time Is Associated With Increased Sensitivity to Autologous Plasma

To evaluate the interaction between viruses and autologous plasma, we took advantage of a pseudovirus-based neutralization assay based on 5, 8, 5, and 6 functional pseudoviruses prepared from plasma at the four time points, respectively (**Table 1**, **Figure 3A**, and **Supplementary Figure 6**). Our results showed that the neutralizing ability of the later plasma to the prophase pseudovirus increased over time (Kruskal–Wallis chi-squared = 25.72, p<0.001). Dunn–Bonferroni testing was used for pairwise *post hoc* comparisons, and the ID50 of the 202009 plasma to pseudoviruses was found to be different from that of 201603 and 201801 plasmas (adjusted p < 0.001, p < 0.01, respectively); the ID50 of the 201811 plasma to pseudoviruses was also different from that of the 201603 plasma (adjusted p < 0.05). Moreover, all plasmas presented low, or no, neutralization to pseudovirus containing the Env gene from sample 202009, possibly by the continuous escape from immune pressure through mutation or glycosylation. Consistent with phylogenetic analysis, PNGS showed an increasing trend over time, indicating that antibody-based selection pressure drove the ongoing viral evolution.

### Broadly Neutralizing Activity of Plasma From Patients

To assess the neutralization breadth of the antibodies in the four sequentially harvested plasma samples, the standardized global HIV-1 reference panel (deCamp et al., 2014) and two highly neutralization-sensitive tier 1A primary isolates widely used in vaccine studies (Qualls et al., 2018) were selected, along with SVA-MLV pseudovirus as the virus control (**Table 2**). Results showed the neutralization breadth to be 100% (with neutralization ID50 > 20 as the cutoff). Also, the geometric mean ID50 titers (GMTs) of neutralization decreased from 800 in 2016 to 404 in 2020. Therefore, our results suggest that plasma harvested from 2016 may contain bNAbs that may serve as a vaccine for HIV-1 prevention and treatment.

**Table 2:** Cross-neutralizing activity of the four time-point samples.

Pseudoviruses	Neutralization ID50			
	201603	201801	201811	202009
X2278_C2_B6^*a*^	141	107	86	71
398-F1_F6_20^*a*^	1009	1020	768	549
TRO.11^*a*^	185	138	106	92
25710-2.43^*a*^	842	758	433	485
CE703010217_B6^*a*^	515	334	243	208
CE1176_A3^*a*^	778	559	383	311
X1632_S2_B10^*a*^	498	391	263	234
CNE55^*a*^	462	314	226	226
CNE8^*a*^	344	244	161	112
BJOX002000.03.2^*a*^	441	366	209	165
CH119.10^*a*^	565	378	241	249
246-F3_C10_2^*a*^	708	506	337	313
SF162.LS^*b*^	9055	10298	9047	8135
MW965.26^*b*^	38900	40600	34400	43700
SVA-MLV	<20	<20	<20	<20
GMTs	800	638	451	404
Breadth	100%	100%	100%	100%

a Standardized global panel of HIV-1 reference strains.

b Two highly neutralization-sensitive tier 1A primary isolates widely used in vaccine studies.

### Decreased Sensitivity of Viral Variants to bNAbs Is Associated With the Evolution of Env Over Time

**Figure 5:**
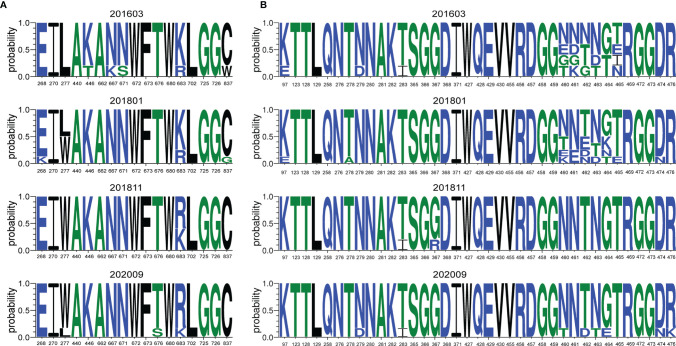
Sequence LOGOs of AA signatures of different bNAbs. The signature sites related with main epitopes or mutation sites associated with neutralization resistance were highlighted. Letter height represents AA frequencies. **(A)** Sequence LOGOs of AA signatures of key determinants associated with sensitivity to 10E8, 2016 to 2020. **(B)** Sequence LOGOs of AA signatures of key determinants associated with sensitivity to VRC01, 2016 to 2020.

We were unable to draw the specificity of broadly neutralizing antibodies from this donor. Nevertheless, the specificities of bNAbs could be indicated by sequence analysis that revealed immune pressure (Ndlovu et al., 2020). To further understand the relationship between sensitivity of viral variants to bNAbs and evolution of Env, we drew the sequence LOGOs of AA signatures of 10E8 and VRC01 in the 4 sequential samples, using HIV-1 antibody-binding sites and the mutation information summarized in the HIV Molecular Immunology Database (https://www.hiv.lanl.gov/content/immunology/index.html), as well as results found in the literature (Bricault et al., 2019) (**Figure 5**).

Residue 683 located in the gp41 transmembrane is ranked 10th among the 10 highest in terms of co-variation with potency and structure to 10E8 (Chuang et al., 2014). Our study showed that residue R683 increased significantly over time, from 21.05% in 2016 to 83.33% in 2020 (**Figure 5A**). The F277W mutation was observed, but the F277I mutation associated with resistance to neutralization of 10E8 (Bricault et al., 2019) was not observed. Residue W277 increased to 83.33% in 2020 after the mutation was observed initially in 2018, suggesting that the F277W mutation may also be related to 10E8 neutralization resistance.

Residue 461 is ranked 6th among the 10 highest in terms of co-variation with CD4bs bNAb VRC01 and structure (Chuang et al., 2014), and variation of the S461N mutation was observed in our study. Bricault reported that D474N and R476K mutations were associated with resistance to neutralization of VRC01 (Bricault et al., 2019), and shifts of D474N and R476K were also observed in our experiment. Residues 460–465 are located in the highly variable region of V5. Our results showed that the residues in this region had been shifting from 2016 to 2020. The S465T mutation increased from 20% in 2016 to 100% in 2020, suggesting that S465T may be associated with VRC01 resistance. To the best of our knowledge, the specific effect of mutation at residue 465 has not been previously reported as indicative of pressure on this epitope and will therefore require additional confirmatory studies.

As shown in **Figure 5**, a considerable variation of amino acid residues with unique specificity over time was observed in relation to the epitopes associated with neutralization resistance to bNAbs.

## Discussion

We followed up on a case with a history of HIV-1 infection for more than 20 years without antiretroviral therapy. During the observation period, the subject had steady CD4 T-cell counts, no clinical signs of AIDS, and slightly increased viral load from 2016 to 2020. The plasma of the donor had 100% neutralization to the global HIV-1 reference panel, showing broadly neutralizing characteristics. Envelope glycoprotein is the binding region of HIV-1-neutralizing antibodies. Sequencing the envelope glycoprotein gene, coupled with an understanding of neutralization phenotype characteristics, can provide a basis for the design of HIV vaccine immunogens and the development of therapeutic neutralizing antibodies. Finally, we longitudinally analyzed the Env genes and neutralization characteristics of the subject from 2016 to 2020 and further explored the possible contribution of signature sites to the resistance of antibodies to neutralization.

With diversification of the Env gene comes diverse antigen stimulation for the immune system, activating naive B cells to induce the maturation of bNAbs (Williams et al., 2021). The results of the phylogenetic tree and genetic distance showed significantly increased host and immunosuppressive effects on virus over time. Despite different evolutionary branches, no significant variation occurred. Our results, which were similar to those of viremic controllers or elite controllers, may also be relevant to quasispecies, the diversity of which remained stable during late virus (Beretta et al., 2020).

Conformational changes and/or glycan shield formation in the Env gp120 play a critical role in HIV-1 immune escape (Behrens et al., 2016). A sequence analysis of variable regions showed that the genetic distance tended to shorten over time. PNGS were fewer, and the amino acid length of each variable region was inconsistent. V1V2 stabilizes the Env spike forming the trimer apex, which plays an essential role in shielding the vulnerable site of the Env spike. The overall variation in Env is usually driven by differences in V1 and V2 loops (Li et al., 2021). Some studies found that the shorter V1V2 region and fewer PNGS were associated with the development of broadly neutralizing antibodies (van den Kerkhof et al., 2013; Wagh et al., 2020). In the present study, longitudinal shortening of the V1 loop may be conducive to the development of bNAbs. The changes of amino acid and glycan motif in the V2, V4, and V5 regions could drive the breadth of bNAbs (Smith et al., 2016). Results showed that the amino acid sequences and PNGS in V4 and V5 regions are increasing and statistically significant. In addition, the amino acid sequence and glycosylation sites in the V2 region showed an increasing trend but no statistical significance. We observed that variants with different modes alternatively appeared over time. The local diversity of variable regions stimulated B cells that brought dynamic somatic hypermutation of BCR, driving the potency and neutralization breadth of bNAbs.

The V1 loop is the main target of autologous bNAb reaction. It is considered potentially protective for vulnerable epitopes, including the V3 loop and the CD4-binding site. Research indicated that long and highly glycosylated V1 regions shield HIV-1 from recognition by V3-directed bNAbs (Silver et al., 2019). V3-crown antibodies show exceptional potency and cross-reactivity in the absence of V1V2 shielding, indicating that distinct conformations of the HIV-1 V3 loop crown can be targeted for broad neutralization (Friedrich et al., 2021). Fewer, or deletion of, PNGS in V1V2 suggested that the V3 loop may be selectively exposed and accessible on the cell surface. The slight fluctuation of genetic distance in the V3 region in our experiment confirmed that the conformational change of Env during virus assembly was inherently complex, as well as dynamic, on the cell surface (Rossignol et al., 2021). The tetrapeptide of the V3 region is the binding site for the host to induce neutralizing antibodies and CTL reaction known to affect viral fusion and entry and determine the usage of co-receptors (Bowder et al., 2018; Behrens et al., 2021). In the process of HIV-1 infection, the co-receptor mainly assists the virus to enter the cell. CCR5 is dominant in the acute infection and asymptomatic phase, whereas CXCR4 is related to progression of the disease. Behrens et al. observed that a low degree of genetic variability and maintenance of functional domains with R5 phenotypes were the hallmarks of V3 region sequences from virologically controlled HIV-infected patients (Behrens et al., 2021). All GPGQ of the tetrapeptides and CCR5-tropic viruses of our results were consistent with long-term non-progressive clinical characteristics of the subject. The highly conserved V3, such as GDIR motif and N332, occurring in different times, suggests that the persistence of the virus into its host during the chronic progression stage of the disease may be subject to functional constraints in addition to the selective pressure of neutralizing antibodies. These unique characteristics of the V3 region may play an important role in buffering the deleterious effect of polymorphisms and increasing genetic robustness (Gasser et al., 2016).

More than 95% of HIV-1 proviruses in the peripheral blood are defective (Imamichi et al., 2020), and Env dysfunction can essentially be explained by the defect of Env protein expression (de Verneuil et al., 2018). In our study, although all of the 59 Env sequences were used to construct pseudoviruses, only 24 functional pseudoviruses could be used for subsequent experiments. HIV-1 bNAbs obtain a large number of somatic mutations, but not all mutations are functionally important. B cells evolve by positive selection to identify boosting candidate immunogens (Swanson et al., 2021). In line with previous observations from viremic controllers or elite controllers (Freund et al., 2017; Kumar et al., 2019), we observed that autologous plasmas can potently neutralize viruses not only from earlier time points but also from concurrent and later time points. The results indicated that the coexistence of the sensitive HIV-1 strain and the effective neutralizing antibody may contribute to controlling viral mutation and disease progression of HIV-1-infected individuals. The neutralizing ability of the later plasma to the prophase pseudovirus increased over time, indicating that the host rapidly evolved neutralizing antibodies against the prophase virus. Deletion of the V1V2 loop or reduction of the glycosylation sites in V1V2 could increase the neutralization sensitivity of autologous plasma and bNAbs (Bontjer et al., 2009; Schorcht et al., 2020). At the 201801 time point, the V1 loop suddenly increased, and the glycosylation sites of the V2 loop increased, explaining, to some extent, the neutralization resistance of autologous plasma at this time point.

For those who have not received antiviral treatment, HIV-1 usually evolves and adapts rapidly by shifting co-receptor tropism and immune escape in the host. In the current study, we observed that all pseudoviruses were resistant to 2G12, PGT121, and PGT135, while the R5 tropism and highly conserved amino acids in the V3 region at the four time points suggested that the virus may have experienced specific amino acid mutations owing to the immune pressure of 2G12, PGT121, and PGT135 in the peripheral circulation before they became the dominant mutant, that is, before 2016 or earlier.

The pseudoviruses at different time points were sensitive to CD4bs antibodies (VRC01, 12A21) and MPER antibody (10E8) but showed neutralization resistance over time, as previously reported (Stefic et al., 2019). The results indicated that escape mutations against CD4bs and MPER epitopes were occurring in the host. Especially for 12A21, several neutralization-resistant variants have appeared, indicating that CD4bs epitopes are evolving rapidly. Our results emphasized that the selective pressure exerted by neutralizing antibodies may be different for different epitopes.

In the process of natural infection, the diversity of viral quasispecies in the host leads to the existence of virus particles with different neutralization sensitivities (Stefic et al., 2017). The sensitivity of pseudoviruses at different time points and even at the same time point to specific bNAbs was significantly different, suggesting the complexity of neutralization phenotype among viral quasispecies. To further analyze the temporal variation characteristics of neutralization sensitivity, we used the same approach as that of a previous study (Rademeyer et al., 2016), namely, detecting the tendency of HIV-1 to drift toward increased neutralization resistance based on genetic evolution. The significant positive correlation of divergence over time and significant positive correlation between IC50 of VRC01, 12A21, and 10E8 and genetic distance indicated that the diversity of functional Env increased over time from 2016 to 2020 and that this diversity was related to neutralization resistance.

Our results are consistent with some previous research (Abela et al., 2019; Wilson et al., 2021). Highly targeted viral evolution, rather than overall envelope diversity, is associated with neutralization breadth (Mabvakure et al., 2019). Our research showed that neutralization sensitivity is also associated with the evolution of the virus that targets the neutralizing epitope, rather than overall envelope diversity. This observation also suggests that future studies on the evolution of functional properties of HIV-1 should be based on the molecular clock, a more accurate witness of viral evolution (Stefic et al., 2019).

In addition, the neutralization specificity of the antibody was subjected to sequence immunological pressure analysis. As far as we know, no publicly reported Env amino acid variation information exists for 12A21; therefore, the results will be reported separately. Here, we only analyzed variation of the key neutralizing epitopes VRC01 and 10E8 over time. We calculated the frequency of key amino acid residues associated with the sensitivity and resistance to individual bNAbs. S461N, D474N, and R476K mutations were observed, but the frequency was small. S465T under selection in the V5 region probably affects susceptibility to VRC01 or associated compensatory mutations, whereas F277W under selection in gp41 probably affects susceptibility to 10E8. Our study attributed immune pressure to escape bNAbs specificities. Indeed, the effects of these mutations on antibody neutralization specificity need further confirmatory research.

In conclusion, through the longitudinal analysis of the Env sequence and neutralization characteristics of an untreated HIV-1 long-term non-progressor with broad neutralization, we describe the genes and variable regions on Env likely modulated in the presence of immune pressure and the evolution of antibodies targeting different epitopes over time. In the absence of selective pressure by antiviral drugs, HIV-1 gradually adapts to human humoral response and neutralizing antibodies in natural infection. These adaptations will inform passive immunization studies and provide new insights that could be useful in the development of immune-based interventions.

Some limitations of the present study should be addressed. First, the subject had been infected for more than 20 years, and the samples of early infection could not be collected. Second, given the iterative evolution of virus and antibody, we were unable to determine whether characteristics were necessary to induce bNAbs or were caused by virus escape reaction. Third, bNAbs used for neutralization sensitivity were limited; therefore, it was unknown whether neutralizing antibodies targeting other epitopes played an essential role in immune responses. Future research dissecting Env epitope specificity and the role of B cells will aid in understanding the coevolution of virus and immune response.

## Data Availability Statement

The datasets presented in this study can be found in online repositories. The name of the repository and accession number can be found in the following: National Center for Biotechnology Information (NCBI) GenBank, https://www.ncbi.nlm.nih.gov/genbank/, OM373318.

## Ethics Statement

The studies involving human participants were reviewed and approved by the Ethics Committee of the National Center for AIDS/STD Control and Prevention, Chinese Centre for Disease Control and Prevention (No. X160729429). The patients/participants provided their written informed consent to participate in this study.

## Author Contributions

LM, KH, and LW conceived the idea and designed the study. LM supervised the research. LW performed the main experiments and wrote the draft manuscript. SL and JH were responsible for the sample and information collection. YD and LH collected and analyzed the data. YH prepared bNAbs. LR and MZ performed the neutralization assay. YL, YF, SJ, and AR helped to analyze the data. SJ, KH, and LM revised the manuscript. All authors contributed to the article and approved the submitted version.

## Funding

This research was funded by the National Major Project of the State Key Laboratory of Infectious Diseases Prevention and Control (Grant No. 2011SKLID102) and the National Natural Science Foundation of China (Grant Nos. 81871694, 81561128006). The funding body had no role in the data collection, analysis, and interpretation of the verbal data, and writing of the manuscript.

## Conflict of Interest

The authors declare that the research was conducted in the absence of any commercial or financial relationships that could be construed as a potential conflict of interest.

## Publisher’s Note

All claims expressed in this article are solely those of the authors and do not necessarily represent those of their affiliated organizations, or those of the publisher, the editors and the reviewers. Any product that may be evaluated in this article, or claim that may be made by its manufacturer, is not guaranteed or endorsed by the publisher.
