# Supplementary material for: Neutralization Sensitivity of HIV-1 CRF07_BC From an Untreated Patient With a Focus on Evolution Over Time
Source: Front Cell Infect Microbiol. 2022 Mar 17;12:862754. doi: 10.3389/fcimb.2022.862754 (PMC8968086; doi:10.3389/fcimb.2022.862754)
Supplement: Supplementary file 1 [file Image_1.pdf]

Supplementary Material

Supplementary Figures

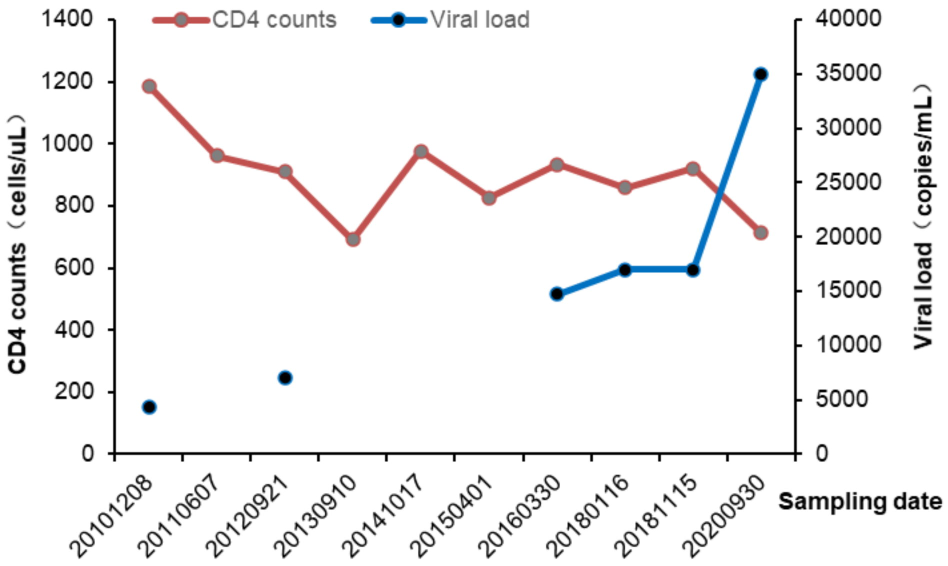

Supplementary Figure 1. Changes over time in CD4 counts and viral load of the patient. CD4 counts and viral load are highlighted in orange line and blue line, respectively. CD4 counts remained at a high level, but declined from December 2010 to September 2020, while viral load trended higher, from March 2016 to September 2020 (Some data for viral load were not available before 2016).

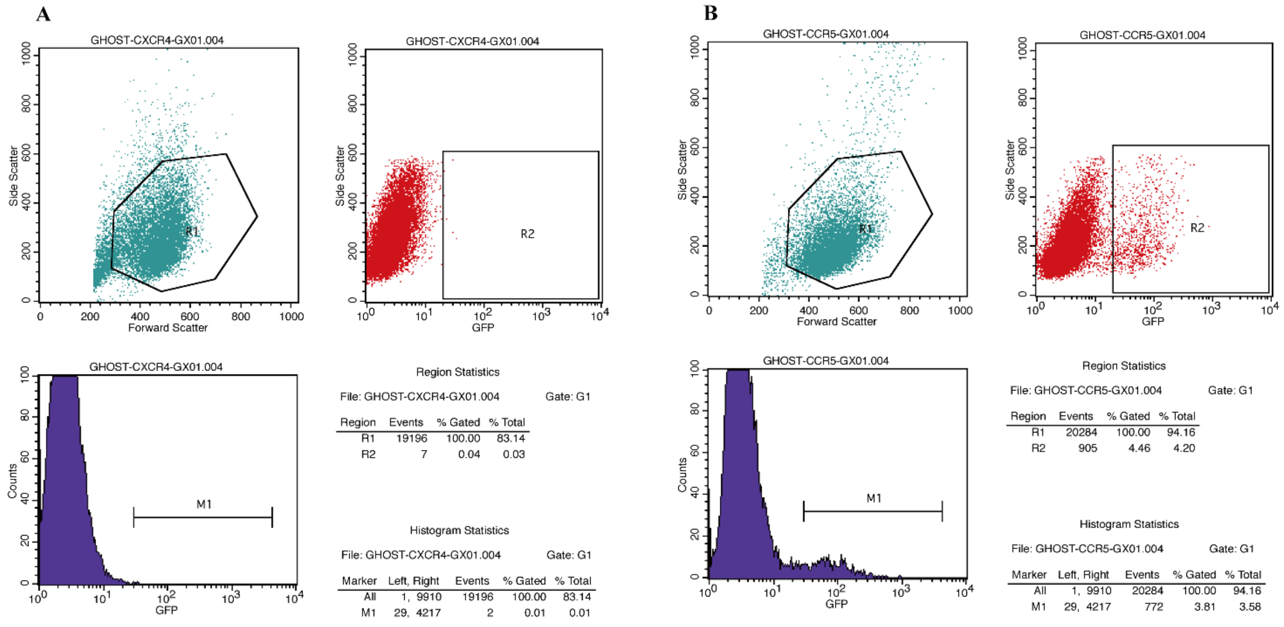

Supplementary Figure 2. Determination of co-receptor tropism for the primary viral isolate GX2016EU01. **(A and B)** Flow cytometry results of primary virus-infected GHOST CXCR4 cells and GHOST CCR5 cells, respectively. Result showed that the primary viral isolate GX2016EU01 induced obvious GFP expression in GHOST CCR5 cells.

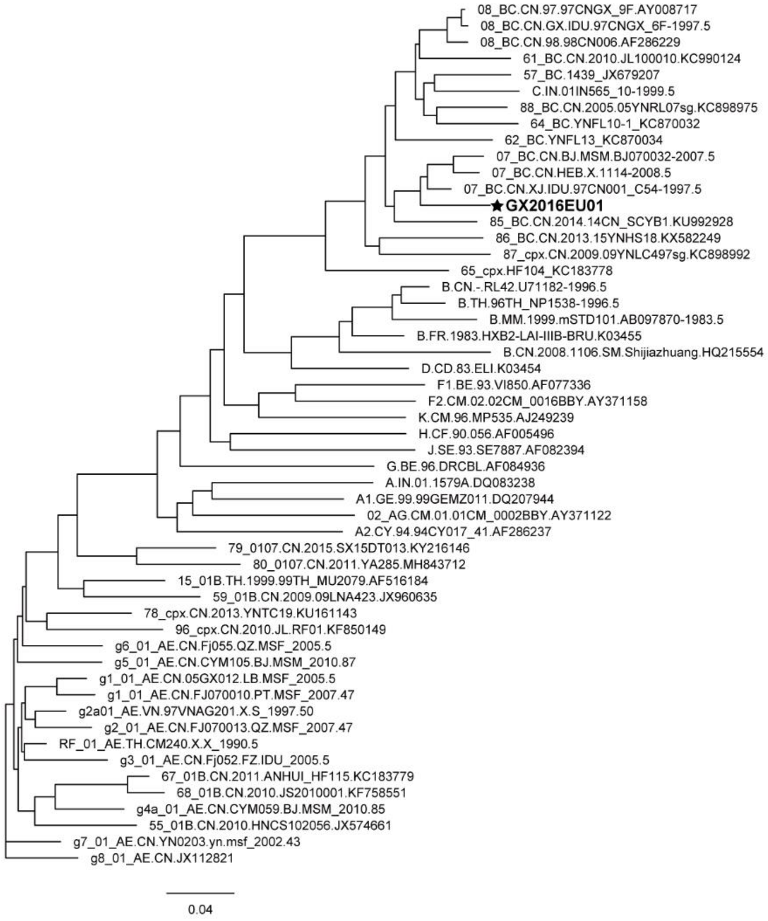

Supplementary Figure 3. Phylogenetic tree analyses of the nearly full-length genome (NFLG) of GX2016EU01. It was constructed with IQtree 1.6.1 using the maximum likelihood method with a general time-reversible model and was visualized by FigTree v1.4.3. GX2016EU01 clustered with the CRF07_BC reference sequences, but forms a monophyletic branch. A solid pentagram (★) marks GX2016EU01, which was also displayed as a bold line.

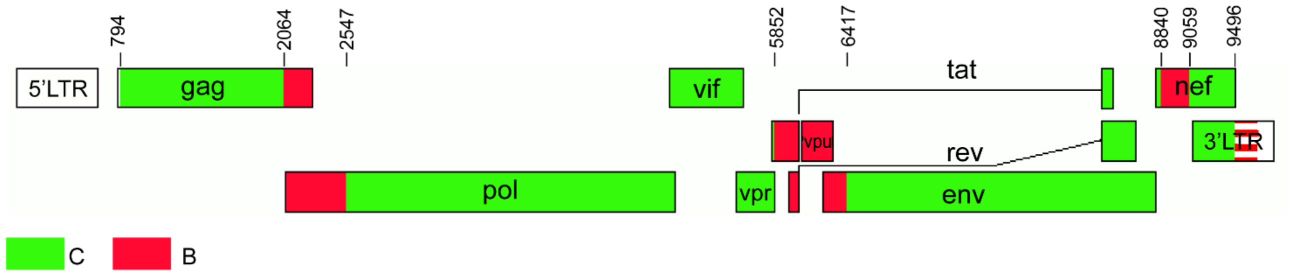

Supplementary Figure 4. Schematic diagram representing the mosaic structure of GX2016EU01 near full-length genome. Genome map of the NFLG sequence of GX2016EU01(based on HXB2 numbering). The mosaic map was generated using the Recombinant HIV-1 Drawing Tool (www.hiv.lanl.gov/content/sequence/DRAW_CRF/recom_mapper. html). Six recombinant breakpoints divided the NFLG into seven segments.

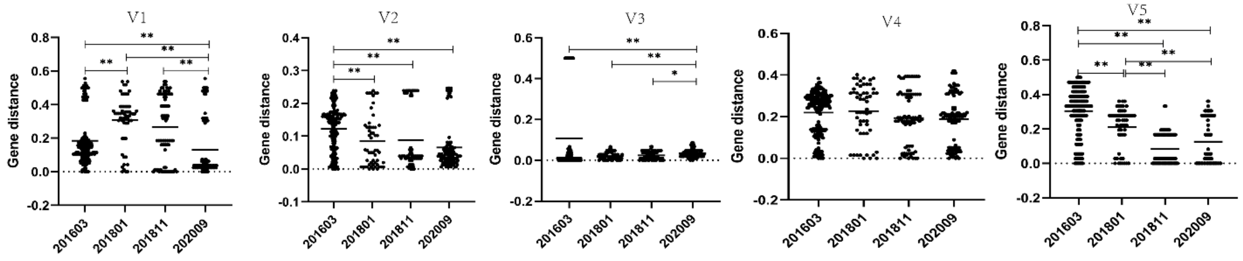

Supplementary Figure 5. Comparison of genetic distance of V1-V5 region among different sampling date. Comparison of V1-V5 region, indicating significant differences in genetic distance of V1, V2, V3, and V5 among different time points. Representative data are shown (* $p < 0.05$; ** $p < 0.01$).

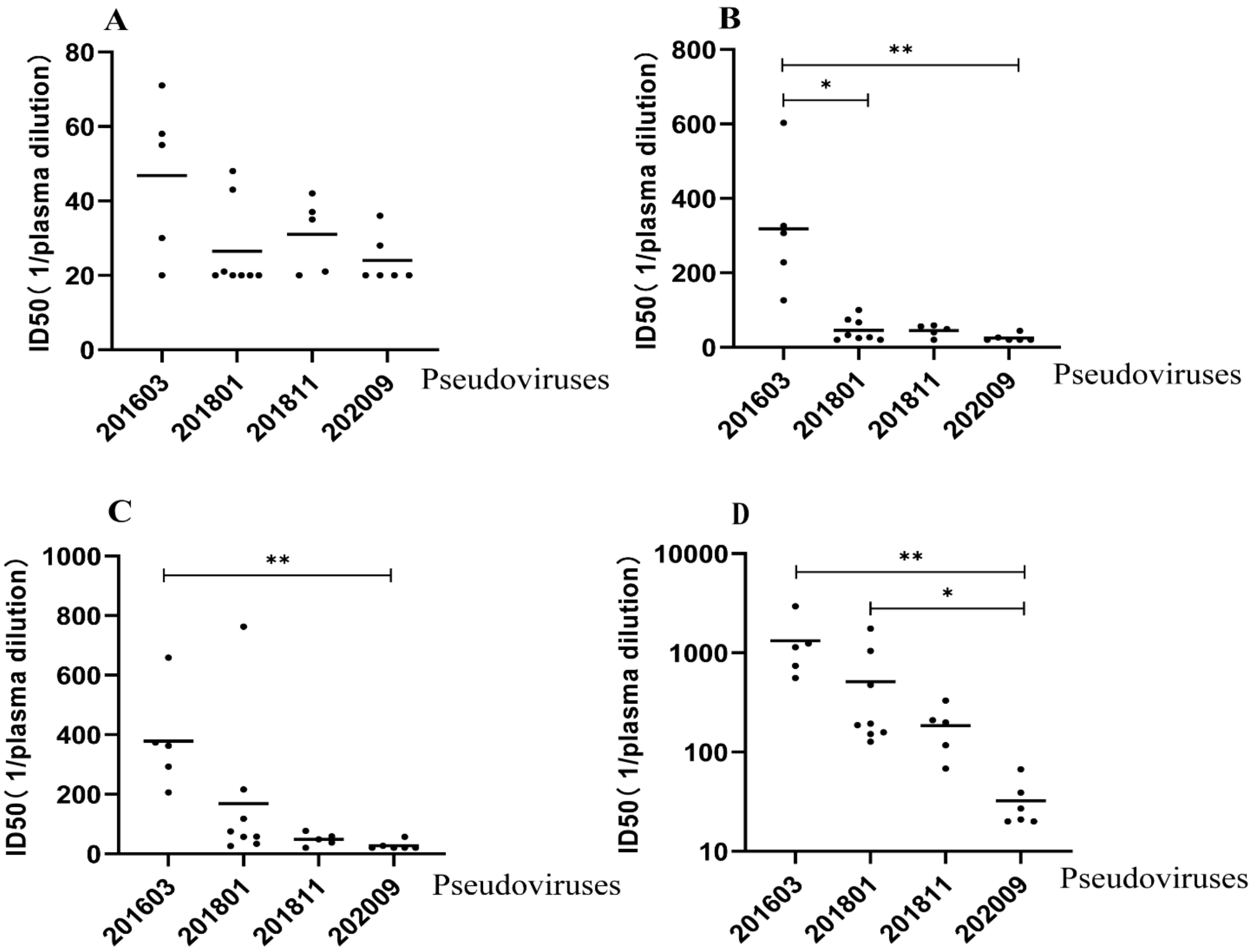

Supplementary Figure 6. Neutralization sensitivity of pseudoviruses to autologous plasma. **(A, B, C, and D)** ID50 values of autologous plasma of 201603, 201801, 201811, and 202009, respectively. Kruskal-Wallis test was performed to assess statistical significance. Representative data are shown (* $p < 0.05$; ** $p < 0.01$).
